# Temporal and Topographical Heterogeneities in Clinical Manifestations of Atopic Dermatitis in China

**DOI:** 10.3390/jcm14030840

**Published:** 2025-01-27

**Authors:** Zheng Li, Huibin Yin, Yu Wang, Shangshang Wang, Yuemeng Wu, Zhuoqiong Qiu, Xu Yao, Chaoying Gu, Wei Li

**Affiliations:** 1 Department of Dermatology, Huashan Hospital, Fudan University, Shanghai 200040, China; zglzdxxyx@163.com (Z.L.); yhb_xiaobin@163.com (H.Y.); 18958161881@163.com (Y.W.); crazygreat@163.com (S.W.); nirvanamay_wu@163.com (Y.W.); qiuzhuoqiong@126.com (Z.Q.); 2 Department of Allergy and Rheumatology, Jiangsu Key Laboratory of Molecular Biology for Skin Diseases and STIs, Hospital for Skin Diseases, Institute of Dermatology, Chinese Academy of Medical Sciences and Peking Union Medical College, Nanjing 210042, China; dryao_xu@126.com

**Keywords:** atopic dermatitis, age, atypical, clinical features, season

## Abstract

**Background/Objective:** Atopic dermatitis (AD) is a chronic inflammatory skin disease characterized by diverse clinical manifestations. However, variations in its clinical presentations across different ages, genders, anatomical sites, and seasons remain incompletely understood. The objective was to explore the clinical heterogeneities of AD using data from the Chinese non-selective registration system. **Methods:** A prospective analysis was conducted on 3829 AD patients enrolled in the Chinese Non-selective Registry for AD (CNRAD) at hospital settings from 2020 to 2022. Demographic profiles; distribution, type, and severity of the skin lesion; laboratory findings; allergic comorbidities; family history; and exacerbating factors were analyzed. **Results:** The male-to-female ratio was 0.92 among adolescent and adult AD patients but increased to 2.11 in elderly AD patients, highlighting an age-dependent gender difference in AD prevalence. Age groups displayed distinct anatomical preferences for lesion distribution, with reduced involvement of the cubital and popliteal fossae in adult and elderly patients. Based on skin lesion characteristics, ten clinical subtypes of AD were proposed. Elderly AD patients exhibited higher severity, compared to adolescence and adult AD patients, with male patients being more severe than females. Elderly AD patients showed a lower proportion of extrinsic type, compared to childhood AD patients. Seasonal change emerged as the most important factor triggering AD flares. **Conclusions:** This study provides new insights into the heterogeneities of AD clinical manifestations in the Chinese population, demonstrating their significant dependence on temporal factors, including age and season.

## 1. Introduction

Atopic dermatitis (AD) is a highly heterogeneous disease [1]. It has been well established that the morphology and distribution of AD lesions change with age [2]. Pediatric AD is characterized by a high prevalence of exudative lesions and seborrhea-like dermatitis, while adult AD exhibits a high occurrence of lichenification and erythroderma [1]. AD with advancing age is linked to a higher incidence of lesions on the buttocks or genitals and a decreased occurrence of lesions on the face and scalp [3]. There are also observed variations in clinical features of AD across different races and (or) regions [1]. Studies conducted in East Asia report a higher prevalence of erythroderma and involvement of the trunk, extensor sites, scalp, and ears [1]. Different phenotypes of AD might be driven by respective underlying mechanisms, or endotypes. Specifically, Asian AD patients demonstrate an increased proportion of T-helper (Th) 22/Th17 immune responses compared to other racial groups [4], while the molecular phenotype of African American AD skin is characterized by reduced Th1 and Th17 responses [5]. However, the heterogeneities in the clinical manifestation of AD patients are still not fully understood.

Over the past few decades, the prevalence of AD in China has been increasing greatly [6,7,8]. It has been noticed that Chinese AD patients exhibit significant variations in clinical manifestation, and Hanifin and Rajka diagnostic criteria are not adequate for the diagnosis of Chinese AD [9,10]. Chinese AD patients usually seek direct medical attention at hospitals without first obtaining a referral from a primary care physician. Consequently, dermatologists in China see a substantial number of AD cases with mild, localized, or atypical skin lesions. Thus, investigation into the AD manifestations in the Chinese medical system would provide a more general view on the heterogeneity of AD.

Previous registrations for AD mostly enrolled moderate-to-severe patients with typical manifestations, with the purpose mainly being for sample collection and (or) treatment evaluation, which usually excludes AD patients with mild, located, or atypical manifestations. To explore the clinical features of Chinese AD patients in a real-world setting, we developed a comprehensive medical record registration system named the Chinese Non-selective Registry for AD (CNRAD) in a hospital-based manner, in which all patients diagnosed with AD were enrolled. Using the CNRAD, we conducted a study with a prospective design, aiming to clarify the heterogeneous clinical features of AD, to summarize different subtypes, and to update the understanding on the clinical manifestation of AD patients in China.

## 2. Methods

### 2.1. Establishment of the Electronic Registry System

CNRAD was incorporated in the medical record registration system of the doctor station of Huashan Hospital in Shanghai, China. Clinical data, including clinical manifestation, diagnosis, complications, and treatment, were collected during the first visit of the patients to build the database. The information in the electronic registry system included onset of AD; history of other atopic diseases, including allergic rhinitis, allergic conjunctivitis, and allergic asthma; family history of atopic diseases; aggravating factors; lesion distribution; types of skin lesions; Eczema Area and Severity Index (EASI) scores; laboratory test results; and past treatments.

### 2.2. Study Design and Patient Enrollment

This was a prospective study which was registered at the Chinese Clinical Trial Registry (ChiCRT Identifier: ChiCTR2000036523). Informed consent was obtained from each subject or his/her parents. All the patients diagnosed with AD who visited the outpatient clinic at the Atopic Dermatitis Center of Huashan Hospital in Shanghai from May 2020 to December 2022 were enrolled. The medical records and laboratory data of 3829 subjects were analyzed.

Diagnosis of AD was made according to the clinical diagnosis of experienced dermatologists in the Atopic Dermatitis Center of Huashan Hospital [10,11,12]. The disease severity was measured using the EASI score. The patients were subdivided according to EASI score: mild < 7, moderate ≥ 7 and <21, and severe ≥ 21. Total immunoglobulin E (IgE) level >240 ng/mL was defined as elevated, according to the range set by the institution. The intrinsic AD was defined by a total IgE less than 240 ng/mL and absence of allergen-specific IgE, while an extrinsic type was defined by a total IgE exceeding 240 ng/mL and (or) being positive for allergen-specific IgE. Serum total IgE level was analyzed using turbidimetric inhibition immunoassay (Siemens, Germany), and eosinophil counts in peripheral blood were obtained from the data of the complete blood count.

### 2.3. Statistical Analysis

Quantitative results were expressed as means ± deviation (SD). The results were analyzed using independent samples, the Mann–Whitney U test, Kruskal–Wallis ANOVA, and Pearson chi-square test. Deviations were considered statistically significant when *p* < 0.05. SPSS version 26.0 (SPSS Inc., Chicago, IL, USA) was used for all statistical analyses.

## 3. Results

### 3.1. Demographic Characteristics

A total of 3829 AD patients were included, of whom the mean age was 30.3 ± 21.3 years at the initial visit. Among the patients, 1.83% were infantile AD patients, 24.55% were childhood AD, 61.58% were adolescence/adult AD, and 12.04% were elderly AD. The low percentage of infantile AD was due to the fact that there was no department of pediatrics in our hospital. Thus, we mainly analyzed the features of AD in adolescents, adults, and the elderly. About 49.14% of the patients developed AD after the age of 18, suggesting that adult-onset AD is common (Table 1).

Overall, there was no difference in the occurrence of AD between males and females (male-to-female ratio, 51.58:48.42). When stratified by age, the percentage of male patients was significantly higher than that of female patients in the group of elderly patients (67.90% vs. 32.10%), whereas in the adolescence/adult group, there were significantly fewer men than women with AD (47.88% vs. 52.12%).

### 3.2. Clinical Manifestation

**Figure 1:**
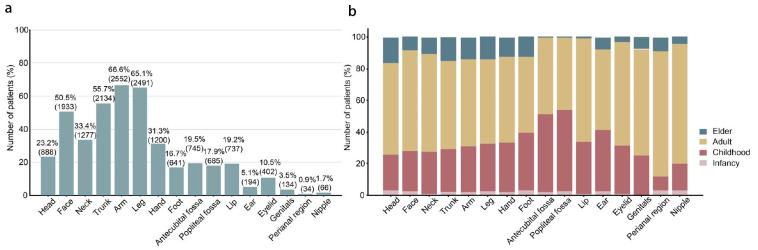
Lesion distribution and its relationship with age. (**a**) Number and percentage of lesion distribution at initial visit. (**b**) Proportion of the lesion distribution according to different age groups.

Evaluation of the skin lesion distribution was first conducted. The top three affected body sites of the patients at initial visit were the arm, leg, and trunk (Figure 1a). The distribution of skin lesions differed among different age groups. In general, childhood AD patients were usually affected in the flexural sites, and the skin lesions of infantile and adult AD patients were more generalized, while elderly AD patients tended to be affected in the extensor sites. AD patients in different age groups also had preferences in lesion distribution: head was the most frequently involved body site for infants, and cubital fossa and popliteal fossa were the most frequently involved body parts for children; cubital fossa, popliteal fossa, eyelid, and lip were the least involved body parts in elderly AD patients (Figure 1b).

### 3.3. Proposed Clinical Subtypes of AD

**Figure 2:**
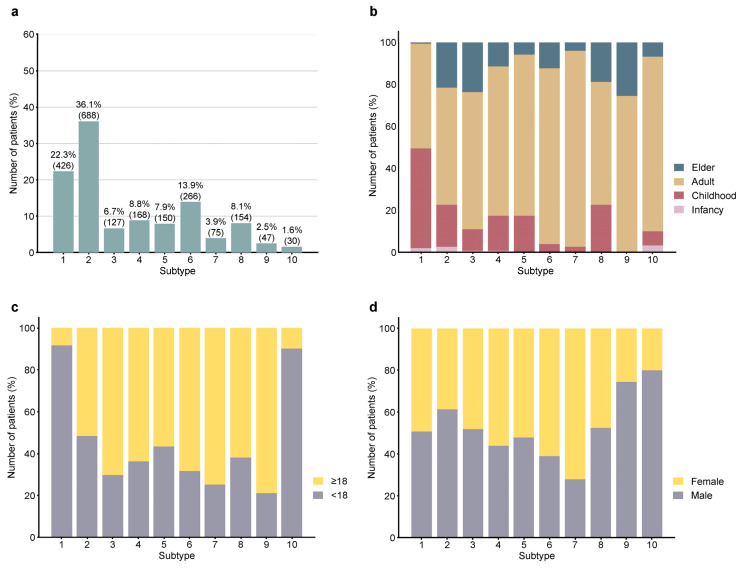
AD subtypes and their relationship with age, age of onset, and gender. (**a**) Number and percentage of the 10 AD subtypes; (**b**–**d**) proportion of the AD subtypes according to different age groups (**b**), onset of age (**c**), and gender (**d**). The mark numbers of 10 AD subtypes representing: (1) the classical flexor type that mainly affects flexure sites of the extremities, neck, and face with lichenized lesions; (2) generalized eczema involving both flexure and extensor sites with polymorphic lesions; (3) reversal distribution type mainly involving the extensor sites; (4) hands and feet eczema; (5) special body site type that affects the lip, nipple, genital, and (or) perianal areas; (6) photosensitivity type that is sensitive to sunlight or have obviously severe lesions in the sun-exposed sites, such as the face, ear, neck, and the back of the hand; (7) seborrheic type that mainly involves the face and back; (8) prurigo nodularis type that presents with prurigo nodularis; (9) amyloidosis type that manifests with amyloidosis; and (10) erythrodermic type where 90% of the body is erythroderma.

Based on the type and location of skin lesions, we proposed 10 subtypes of AD, which were recorded in CNRAD: (1) the classical flexor type that mainly affects flexure sites of the extremities, neck, and face with lichenized lesions; (2) generalized eczema that involves both flexure and extensor sites with polymorphic lesions; (3) reversal distribution type that mainly involves the extensor sites; (4) hands and feet eczema where only the hands and feet are affected; (5) special body site type that affects the lip, nipple, genital, and (or) perianal regions; (6) photosensitivity type that is sensitive to sunlight or have obviously severe lesions on the sun-exposed sites, such as the face, ear, neck, and the back of the hand; (7) seborrheic type that mainly involves the face and back; (8) prurigo nodularis type that presents with prurigo nodularis; (9) amyloidosis type that manifests mainly with amyloidosis; and (10) erythrodermic type where 90% of the body is erythroderma. The proportion of different clinical subtypes was assessed. Generalized eczema had the highest incidence (36.06%), followed by the classic flexor type (22.33%) (Figure 2a). The relationships between the subtypes and other factors, such as gender, age, and severity, were also analyzed. The results showed that the erythrodermic-type AD had the highest EASI score and mainly occurred in males. Elderly AD seldom appeared as the classical flexor type affecting only the flexor side but had increased proportions of the reversal distribution, prurigo nodularis, and amyloidosis types (Figure 2b). Similar features were found in adult-onset AD, which had increased proportions of the photosensitivity type and seborrheic type of manifestation (Figure 2c). Interestingly, the photosensitivity type and seborrheic type were more common in women (Figure 2d).

### 3.4. Disease Severity

The average EASI score was 7.86 (±8.02) for all the AD patients enrolled, and severe AD accounted for 7.29% of total patients (Table 1). The EASI scores of male patients were higher than those of female patients (EASI score, 9.12 vs. 6.53). The EASI scores were also compared among different age groups. The result showed that the adolescence/adult AD (EASI score, 7.43 ± 8.33) was less severe compared to elderly AD (EASI score, 9.52 ± 7.78), and adult-onset AD had the least severity. Moreover, the correlations of disease severity with comorbid allergic diseases and family history were also analyzed. The EASI scores were higher in the AD patients with a history of allergic asthma and those with a family history of allergic diseases for grandparents or parents.

### 3.5. Aggravating Factors

Aggravating factors, including seasonal variation, environmental temperature, sweating, and sunlight exposure, were recorded, and the results of the analysis are shown in Table 2. Two thousand two hundred and eighty-four (60.84%) patients reported seasonal changes as the aggravating factor inducing the flares. Both summer (34.71%) and winter (23.49%) were the most reported seasons during which the disease became worse. The least common season for AD exacerbation was autumn, as only 143 (3.81%) patients reported that their dermatitis got worse in autumn. The infant-, elderly-, and adult-onset AD were less influenced by season, compared to other age groups. The relationship between clinical subtypes and seasons was also analyzed. There was a large proportion of patients in the classical flexor type and photosensitivity type exacerbated during the summer months (48.24% and 49.62%, respectively), and the amyloidosis type and reversal distribution type tended to become aggravated in the winter (36.17% and 32.28%, respectively). More than half of the hand and feet eczema, special body site type, and erythroderma type had no correlation with season.

One thousand two hundred and seventy-five (69.03%) patients reported that the symptoms were aggravated by the sudden rising of environmental temperatures. The adult-onset AD was less influenced than early-onset AD (who developed AD prior to the age of 18). Additionally, the patients who were aggravated under the circumstance of hot temperatures reported an increased proportion of sweating malfunctions (44.24% vs. 30.60%).

One thousand six hundred and forty-eight (43.82%) patients reported that the symptoms were aggravated when exposed to sunlight, and the majority of them were also aggravated during the summer, with the skin lesions mainly involving the exposure area, such as the head, face, and neck. Less than half of the proportion of patients (39.36%) reported sweating malfunction, and the patients who were aggravated during the summer reported an increased proportion of sweating malfunction. Furthermore, the patients with lesions on the face had a higher incidence of sweating malfunction than those with skin lesions in other areas. It was interesting to note that with the increase in age, there was a decrease in the proportion of patients influenced by sunlight exposure, although without statistical significance.

### 3.6. Comorbid Allergic Diseases and Family History

Allergic comorbidities, a feature of AD, including allergic rhinitis, allergic asthma, and allergic conjunctivitis, were assessed next. More than half of the patients (62.69%) had allergic comorbidities, among whom 76.38% had a single comorbidity, and the rest (23.62%) had multiple comorbid atopic diseases. Among the concomitant single allergic diseases, allergic rhinitis was the most frequently reported (43.11%), which was much higher than allergic asthma (3.48%) and allergic conjunctivitis (1.29%). Nearly 62.76% of the patients had a family history of at least one allergic disease, and AD was the most frequently reported disease, followed by allergic rhinitis (Table 3).

### 3.7. Laboratory Findings

**Figure 3:**
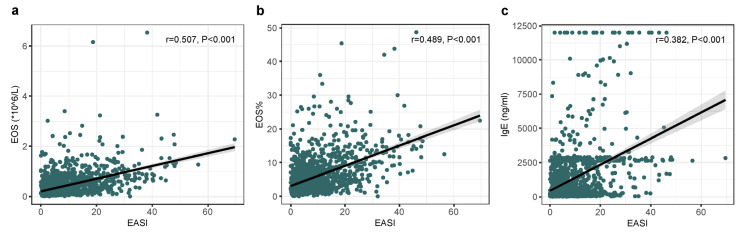
Correlation of EASI with eosinophils and IgE. Scatter plots of EASI versus the number of eosinophils (**a**), the percentage of eosinophils (**b**), and IgE level (**c**).

The laboratory findings are shown in Table 1, including the serum levels of total IgE and eosinophil count. There was a higher percentage of extrinsic (58.81%) AD than intrinsic AD (41.19%). With the increase in age, the proportion of extrinsic AD showed a decreasing trend (70.65% for childhood AD, 58.18% for adolescence/adult, and 51.61% for elderly). The incidence of extrinsic type in adult-onset AD was significantly lower, compared to the patients who developed the disease before the age of 18 (46.47% vs. 76.82%), and the eosinophil count of adult-onset AD was also lower (338 × 10^6^ vs. 555 × 10^6^). The correlation between serum total IgE, blood eosinophil count, and EASI score were then assessed, and the results showed that the increase in serum total IgE level and blood eosinophil count were significantly positively associated with the EASI score (Figure 3).

## 4. Discussion

Heterogeneity is one of the fundamental features of AD, encompassing clinical [1,2,3,4,5,6,7,8,9,10,11,12,13] and molecular phenotypes [14], inflammatory mechanism [15,16], and responses to treatment [17]; however, the current knowledge on the heterogeneity of AD still remains elusive. By employing an electronic registry system for non-selective outpatients in China and a prospectively designed study, we tackled the heterogeneity in clinical manifestations of Chinese AD, which was attributed largely to variations in age and gender. There were a considerable number of adult-onset patients that showed distinct clinical features and lacked comorbid atopic diseases or a family history in China. We proposed 10 distinct classifications of clinical manifestations, according to the type and location of skin lesions, for a comprehensive understanding of the atypical manifestations of AD. Climate- and environment-related factors, such as season and temperature, were related to the flares of AD. Thus, our study enriched the understanding of the heterogeneity of AD in China by investigating the clinical features based on the Chinese medical system.

The clinical manifestation of AD described by classical diagnosis criteria mainly have “typical morphology and distribution” [18], which restricts the recognition and diagnosis of AD, especially in China. There are a large proportion of AD patients with manifestations that are not typical [19,20,21], for example, atypical morphology, including the seborrheic or erythrodermic type of skin lesion, prurigo nodularis, and amyloidosis [22,23], and atypical locations, such as the hands/feet and genital/perianal region [19]. Adult-onset AD and elderly AD can also be deemed as a kind of atypical AD, as AD was once thought of as a disease of children [24]. We took advantage of the Chinese medical system and developed a non-selective registry (CNRAD). The CNRAD is incorporated in the doctor station, and it is easy to use and facilitates the efficient collection of a large amount of information, enabling a more comprehensive evaluation of AD. The CNRAD is also useful for the future evaluation of the treatment and long-term management of the patients.

Our study revealed that there were a large number of adult-onset AD patients in China and provided insight into this particular AD subset. Our results showed that adult-onset AD patients exhibited lower disease severity, which is similar to a multicenter study conducted in Italy [25]. However, another study reported no significant difference in disease severity [26]. Additional research is warranted to validate these findings. Consistent with previous research [27], our findings also showed that a significant proportion of adult-onset AD patients did not have a personal or familial background of atopy or elevated IgE levels, thereby posing challenges in distinguishing AD from alternative diagnoses. Additionally, our study revealed that these patients exhibited lower eosinophil counts, compared to early-onset AD patients. They also displayed reduced susceptibility to external environmental factors, such as seasonal variations and sun exposure. The unique features of adult-onset AD indicated that they might have a different pathogenesis compared to early-onset AD, and further studies are needed.

Elderly AD, the recently identified fourth group of AD stratified by age, has been attracting increasing interest worldwide, although there is a lack of knowledge regarding the clinical manifestation, underlying endotype, and pathogenesis. Our results aligned well with previous reports, showing that the involvement of the cubital and popliteal fossae was typically less pronounced in elderly AD [28]. The majority of elderly patients in our study developed the disease after becoming 60 years old (67.17%). Furthermore, our study revealed that elderly male patients exhibited higher disease severity and were more prone to developing atypical skin lesions, such as prurigo nodularis. Notably, a higher prevalence of male patients was observed in elderly AD, whereas the prevalence of AD was slightly higher in female during adolescence and beyond. The gender disparities observed within different age groups suggest a potential association between sex hormones and the underlying mechanisms of AD, especially for the elderly.

To summarize the heterogeneity in the morphology and distribution of skin lesions of Chinese AD, we proposed 10 subtypes that were featured by body sites (classical flexure type, generalized eczema, reversal distribution type, hands and feet eczema, and special body site type), lesion types (prurigo nodularis, amyloidosis, and erythrodermic type), or possible pathogenesis (seborrheic type and photosensitivity). This classification did not intend to make a simple thing complex but to cover the diverse manifestations of AD and include those “atypical” patients. In our registry, there were high proportions (53.30%) of the types apart from the classical flexure type and generalized eczema, indicating a high number of “atypical” AD patients in China. It is still difficult for a few AD patients to be included in the classification, particularly for individuals presenting with mild and subtle lesions. Additionally, a newly proposed subtype, psoriasis dermatitis, has emerged, highlighting shared characteristics between AD patients and those with psoriasis [29]. Thus, our classification still needs to be refined in the future. The mechanisms underlying different types of AD might be different, and extensive explorations will be needed in the future.

It has been reported that environmental factors have an impact on the onset and reoccurrence of AD [30]. Our study revealed that seasonal changes significantly exacerbated the symptoms of Chinese AD patients. Particularly, there were a large proportion of AD patients who became worse during the summer and (or) winter. A survey conducted in Korea, a country with a climate similar to that of China, revealed comparable findings [31]. Similarly, a study conducted in the United Kingdom highlighted that hot weather and sweating are the predominant exacerbating factors [32]. Furthermore, our study revealed a correlation between the exacerbation of symptoms in patients with AD in the summer and an elevated occurrence of abnormal sweating. The possible mechanism might be the abnormality in the autonomous nervous system of AD patients. Additionally, it was observed that symptoms further deteriorated upon the sudden rising of temperatures. These findings strongly suggest that both temperature and sweating play a role in the aggravation of AD during the summer. Conversely, decreased humidity and dry skin may be the causes of aggravation during the winter. Further investigation is warranted to gain a comprehensive understanding of the underlying mechanism behind the seasonal intensification of AD.

There were several limitations to be considered when interpreting our results. Our study was based on a single center in an economically developed city, where the majority of patients visiting were in a relatively favorable financial condition. The sample size in the current study was relatively small when compared to the overall Chinese population. We did not mention skin infections in AD patients, as their increased susceptibility to viral and bacterial infections [33,34], resulting from compromised skin barrier function, may confound the assessment of skin lesion morphology due to potential co-infections. Consequently, multicenter studies incorporating larger samples across all age groups, socioeconomic backgrounds, and racial demographics are necessary to enhance the characterization of the clinical and epidemiological profiles of atypical AD.

## Figures and Tables

**Table 1:** Demographics and clinical characteristics.

	Values	Available Sample Size
Gender		n = 3829
Male, n (%)	1975 (51.58%)	
Female, n (%)	1854 (48.42%)	
Age at initial visit (SD), y	30.3 (21.3)	n = 3829
Infancy (<2 years), n (%)	70 (1.83%)	
Childhood (2–12 years), n (%)	940 (24.55%)	
Adult/Adolescence (12–60 years), n (%)	2358 (61.58%)	
Elder (>60 years), n (%)	461 (12.04%)	
Age of onset (SD), y	22.3 (22.1)	n = 3824
<18 years, n (%)	1945 (50.86%)	
≥18 years, n (%)	1879 (49.14%)	
Disease duration (SD), m	96.2 (106.3)	n = 3824
EASI ^†^ (SD)	7.86 (8.02)	n = 3828
Mild (<7), n (%)	2350 (61.39%)	
Moderate (7–21), n (%)	1199 (31.32%)	
Severe (≥21), n (%)	279 (7.29%)	
Male (SD)	9.12 (8.82)	n = 1974
Female (SD)	6.53 (6.84)	n = 1854
Infancy (SD)	9.13 (7.91)	n = 70
Childhood (SD)	8.03 (7.22)	n = 939
Adult/Adolescence (SD)	7.43 (8.33)	n = 2358
Elder (SD)	9.52 (7.78)	n = 461
Age of onset < 18 years (SD)	9.27 (9.02)	n = 1944
Age of onset ≥ 18 years (SD)	6.41 (6.54)	n = 1879
VAS ^‡^ (SD)	4.80 (2.35)	n = 3827
Eosinophils		n = 1366
Eosinophil count (SD), ×10^6^/L	422 (487)	
Eosinophil % (SD)	5.72% (5.53%)	
IgE ^§^		n = 1459
>240 ng/mL, n (%)	858 (58.81%)	
≤240 ng/mL, n (%)	601 (41.19%)	

Values are presented as number (%) or mean ±SD. ^†^: EASI, Eczema Area and Severity Index; ^‡^: VAS, visual analogue scale; ^§^: IgE, immunoglobulin E.

**Table 2:** Aggravating factors.

Aggravating Factor	Number of Patients (%)
Seasonal variation	n = 3754
Aggravated with season, n (%)	2284 (60.84%)
Aggravated in spring, n (%)	189 (5.03%)
Aggravated in summer, n (%)	1303 (34.71%)
Aggravated in autumn, n (%)	143 (3.81%)
Aggravated in winter, n (%)	882 (23.49%)
Aggravated with season change, n (%)	156 (4.16%)
Sweating	n = 3397
Normal, n (%)	2060 (60.64%)
Increase, n (%)	628 (18.49%)
Decrease, n (%)	709 (20.87%)
Rising temperature	n = 1847
Aggravated, n (%)	1275 (69.03%)
Sunlight	n = 3761
Aggravated with sunlight, n (%)	1648 (43.82%)

Values are presented as a number (%).

**Table 3:** Number of patients with comorbid allergic diseases and a family history of atopic conditions.

	Number of Patients (%)
History of atopic diseases	n = 3417
Presence of comorbid allergic disease, n (%)	2142 (62.69%)
AR ^†^, n (%)	1473 (43.11%)
AA ^‡^, n (%)	119 (3.48%)
AC ^§^, n (%)	44 (1.29%)
AR + AA, n (%)	278 (8.14%)
AR + AC, n (%)	144 (4.21%)
AA + AC, n (%)	8 (0.23%)
AR + AA + AC, n (%)	76 (2.22%)
Family history	n = 3394
Positive family history, n (%)	2130 (62.76%)
AD ^¶^, n (%)	1284 (37.83%)
AR, n (%)	887 (26.13%)
AA, n (%)	134 (3.95%)
Grandparents, n (%)	256 (7.54%)
Parents, n (%)	1534 (45.20%)
Brothers and sisters, n (%)	161 (4.74%)
Offspring, n (%)	404 (11.90%)
Grandchildren, n (%)	61 (1.80%)

Values are presented as a number (%). ^†^: AR, allergic rhinitis; ^‡^: AA, allergic asthma; ^§^: AC, allergic conjunctivitis; ^¶^: AD, atopic dermatitis.

## Data Availability

All data shown in the figures and tables and additional raw data are available upon request from the corresponding author.

## Abbreviations

AA	allergic asthma
AC	allergic conjunctivitis
AD	atopic dermatitis
AR	allergic rhinitis
CNRAD	Chinese Non-selective Registry for AD
EASI	Eczema Area and Severity Index
IgE	immunoglobulin E
SD	standard deviation
Th	T-helper
VAS	visual analogue scale
